# DeepCentering: fully automated crystal centering using deep learning for macromolecular crystallography

**DOI:** 10.1107/S160057751900434X

**Published:** 2019-06-03

**Authors:** Sho Ito, Go Ueno, Masaki Yamamoto

**Affiliations:** a ROD (Single Crystal Analysis) Group, Application Laboratories, Rigaku Corporation, 3-9-12 Matubara-cho, Akishima, Tokyo 196-8666, Japan; b Graduate School of Life Science, University of Hyogo, 3-2-1 Kouto, Kamigori, Ako, Hyogo 678-1205, Japan; c RIKEN SPring-8 Center, 1-1-1 Kouto, Sayo, Hyogo 679-5148, Japan

**Keywords:** automated crystal centering, deep learning, fully automated structure determination

## Abstract

High-throughput protein crystallography using a synchrotron light source is an important method used in drug discovery. Beamline components for automated experiments including automatic sample changers have been utilized to accelerate the measurement of a number of macromolecular crystals. However, unlike cryo-loop centering, crystal centering involving automated crystal detection is a difficult process to automate fully. Here, DeepCentering, a new automated crystal centering system, is presented. DeepCentering works using a convolutional neural network, which is a deep learning operation. This system achieves fully automated accurate crystal centering without using X-ray irradiation of crystals, and can be used for fully automated data collection in high-throughput macromolecular crystallography.

## Abstract 2

A fully automated crystal centering system using deep learning is presented. Using this system, a fully automated crystal structure determination pipeline has also been developed.

## 1. Introduction   

Automated experimental environments in macromolecular X-ray crystallography (MX) have been developed at many synchrotron light sources and have greatly contributed to accelerating the determination of crystal structures. Automated diffraction experiments require an accurate and credible centering system of protein crystals. Here we present DeepCentering, a novel centering system based on automated sample recognition by digital image processing using a convolutional neural network (CNN).

Existing automated centering systems developed at various synchrotron facilities are roughly categorized into three types in terms of sample recognition: digital photo-image processing; X-ray diffraction (so-called raster scanning); and others, such as ultraviolet (UV) or infrared (IR) irradiation (Asanov *et al.*, 2001 ▸; Bourgeois *et al.*, 2002 ▸; Pohl *et al.*, 2004 ▸; Vernede *et al.*, 2006 ▸; Chavas *et al.*, 2011 ▸; Madden *et al.*, 2011 ▸). Among these methods, image-processing-based systems are by far the simplest method as they do not require specific apparatus or X-rays, just an image-processing program such as feature extraction. However, the accuracy of the sample recognition using this method is generally lower than that of manual centering by human eye.

Diffraction-based crystal centering (Song *et al.*, 2007 ▸) has been implemented at many synchrotron MX beamlines. However, this method requires a whole scan over the cryo-loop, which is time-consuming even when using high-frame-rate detectors such as the EIGER series (DECTRIS). In particular, the scan time increases when using the larger cryo-loop compared with the beam size. Moreover, diffraction-based centering cannot avoid radiation damage even though the incident beam is attenuated. Furthermore, the dose effect is a severe problem for protein crystals when using room-temperature crystallography.

The second-harmonic-generation-based crystal centering system (Kissick *et al.*, 2013 ▸) is a dose-free method that provides high centering accuracy. The system is able to detect only chiral crystals that are suitable for detecting protein crystals. However, this system requires additional apparatus such as femtosecond IR lasers.

While the digital photo-image processing method has many advantages, the development of an effective automated centering system has proved difficult. Some existing systems work by subtracting background images to detect the outline of cryo-loops and use simple feature extraction to detect crystals in the images (Lavault *et al.*, 2006 ▸). However, these methods are unable to cope with images with a significant background change. The accuracy of the crystal detection also decreases when the boundary between the crystal and mother liquor is ambiguous. However, there have been recent technological breakthroughs in object detection techniques in digital image processing, most of which utilize CNNs. The method has been adopted in various fields, not only in object detection but also in crystallography, particularly in X-ray serial crystallography (Ke *et al.*, 2018 ▸) and crystallization (Bruno *et al.*, 2018 ▸). DeepCentering was developed based on a CNN to apply the method to cryo-loop and crystal detection for automated centering, and is expected to provide a novel system based on the nonconventional object detection algorithm which is robust against background changes and cases where the boundary between the crystal and mother liquor is ambiguous.

DeepCentering has been implemented at SPring-8 MX beamlines and tested by various loops and crystals, and has shown a high success rate of centering crystals at the beam center. DeepCentering was achieved using accurate automated crystal centering without X-rays or an additional apparatus. This system is applicable to fully automated diffraction data collection and structure determination.

## 2. Methods   

### 2.1. Development of cryo-loop and crystal detecting programs   

Several deep learning frameworks are currently available, such as Tensorflow, Chainer, Caffe and MxNet. DeepCentering was developed utilizing TensorFlow Object Detection API (Huang *et al.*, 2017 ▸) because it is easy to use and the framework was actively developed and is frequently upgraded worldwide. We developed two programs to detect the cryo-loop and crystal (hereafter ‘LoopDetector’ and ‘CrystalDetector’, respectively), as components of DeepCentering. Both detectors were trained using Single Shot MultiBox Detector (Liu *et al.*, 2016 ▸), a robust CNN method. To develop LoopDetector, 6031 cryo-loop images were prepared as training data taken for mail-in data collection at SPring-8 BL26B2 (Okazaki *et al.*, 2008 ▸; Murakami *et al.*, 2012 ▸). We marked the cryo-loop area in each image to highlight the cryo-loop position for the TensorFlow Object Detection API training program. In order to avoid overfitting, we first split the 6031 cryoloop images into a training set and a test set using 24 batch numbers. Both sets of loss functions were monitored. Learning was terminated at an epoch number of 50, which is a sufficiently small epoch number where the loss function difference between the learning set and the test set is not so large

**Figure 1:**
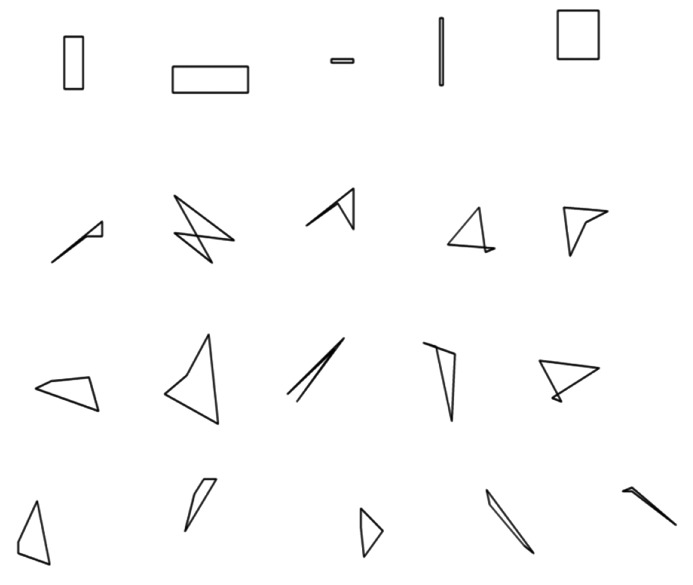
Examples of training images used in CrystalDetector. A total of 418 images were used as training models. All images were generated by Python PIL library.

For CrystalDetector, real crystal images in the cryo-loops were initially used as training data; however, CrystalDetector trained using these data was unable to detect crystals in the images sufficiently. Therefore, various polygon patterns such as triangular and tetragonal generated using the Python PIL library were used as training data instead (Fig. 1 ▸). In total, 418 images were used as training data. Although the number of images for the training data was minimal from the viewpoint of deep learning, the practical CrystalDetector was appropriately trained due to the nature of the protein crystals, as their shapes matched the prepared training data. Since the boundaries between the crystal and mother liquor of the real crystal images were ambiguous, polygon patterns with clear edges were suitable for training data within the limited training data. Unlike in the case of LoopDetector, when CrystalDetector overfits, it incorrectly recognizes the entire image as a crystal, so it was possible to immediately judge that the learning was not successful. Therefore we trained with multiple epoch numbers without splitting all the images into training and test sets. The validation of the result was actually performed using the newly prepared crystal images. The batch number was also 24 for CrystalDetector. As a result, when the epoch number is 8, the detection result is much better than for the cases of other epoch numbers, so we selected this epoch number to create a crystal detector.

### 2.2. Sample detection testing and fully automated structure determination by DeepCentering   

DeepCentering was implemented and all test experiments were performed at BL26B2, which has standard bending-magnet beamline design at SPring-8 (Ueno *et al.*, 2006 ▸). The beamline was aimed to acquire high-throughput data from vast amounts of macromolecular crystals, applicable for routine data collection such as ligand screening. The end­station is equipped with a horizontal goniometer with an air-bearing spindle unit and a three-dimensional pulse motor stage for crystal positioning. A digital microscope coaxial to the X-ray beam with an automatic zooming function is available for sample observation and capturing VGA 24-bit digital color images. The typical beam size and photon flux at the sample are 80 µm × 90 µm, in horizontal and vertical full width at half-maximum (FWHM), and 1.5 × 10^11^ photons s^−1^ using an X-ray energy of 12.4 keV.

**Figure 2:**
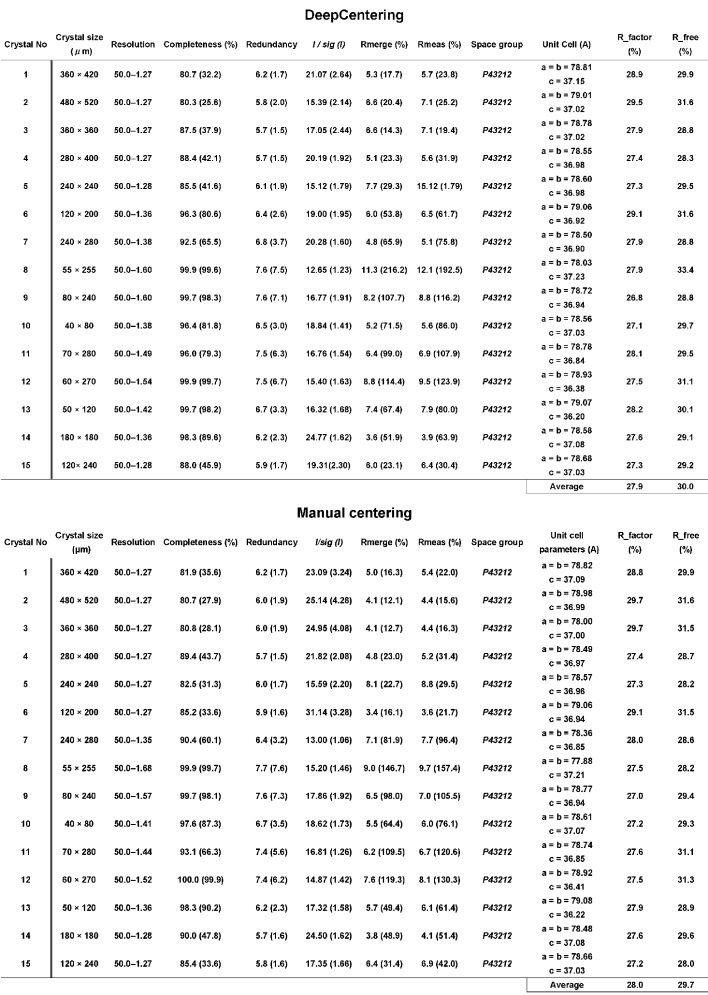
Data processing and structure refinement statistics of lysozyme crystals by DeepCentering and manual centering. Data collection covered 180° of the ω axis, and the rotation angle and exposure time were 0.5° and 0.5 s for each frame, respectively. All data were measured using a 1 Å wavelength at 150 mm camera distance (edge resolution of the detector = 1.58 Å), and shutterless measurement.

First, a test experiment was conducted for cryo-loop detection using LoopDetector with 100 samples consisting of two types of loops: nylon and lytholoop. Second, the crystal detection test was performed as follows. Crystals were kindly provided by beamline users, and the images were manually captured by the digital microscope at the beamline. The total number of images was 960. Finally, the test images were processed using CrystalDetector to evaluate success rates, and the whole centering flow that covers the loop centering and the crystal detection using lysozyme crystals was tested. Afterwards, the automated system proceeded to diffraction data collection, followed by data processing using *KAMO* (Yamashita *et al.*, 2018 ▸) and a structure determination pipeline locally developed based on *Dimple* (Winn *et al.*, 2011 ▸). Data processing and structure refinement statistics were compared with diffraction data from the same protein crystals with manual centering. All experimental statistics are shown in Fig. 2 ▸.

### 2.3. Flow of the automated centering procedure   

**Figure 3:**
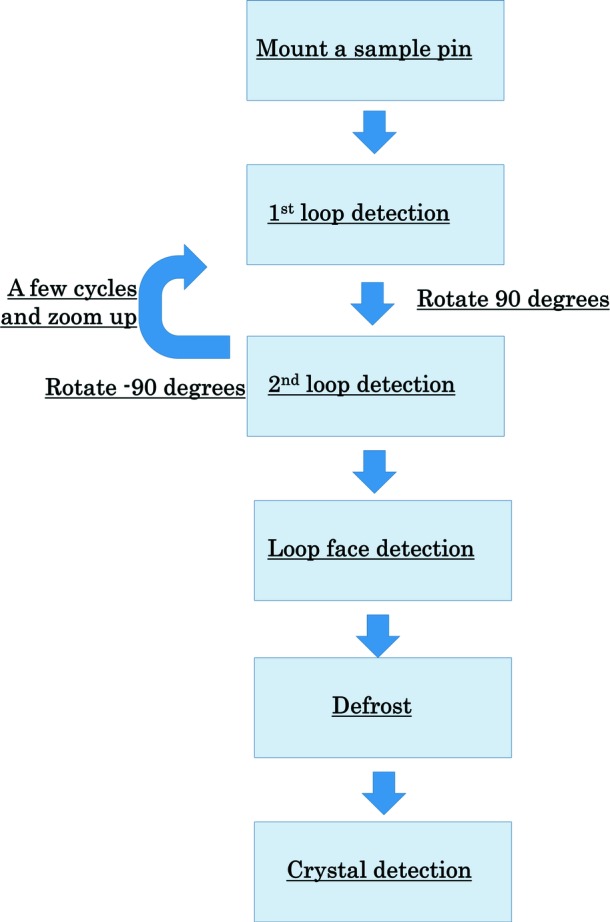
Flow of DeepCentering. LoopDetector is used a few times until the loop position is converged. Before CrystalDetector operates, an automatic defroster system pours liquid nitrogen onto the sample to remove frost stuck to the cryo-loop.

The automated centering procedure using DeepCentering was performed as follows (Fig. 3 ▸). Remarkably, this automated centering system does not require initial processing of images or a digital microscope, such as binarization of captured images and optimization of brightness and contrast. (1) Cryo-loop centering was performed from two directions with LoopDetector. Firstly, an image was taken at a reference spindle angle, and then the goniometer spindle was rotated by +90° from the first angle and a second image was taken. (2) The zoom ratio of the coaxial camera was changed to higher magnification for precise centering, and the sequence was repeated from step (1) until the loop position was converged. (3) The cryo-loop face was oriented perpendicular to the X-ray beam, so that the width of the detected object was maximized, using the least-squares fitting method. (4) To improve results of crystal detection, we installed a defroster system whereby the cryo-loop is sprayed with liquid nitro­gen to remove frost. (5) The crystal on the cryo-loop was detected using CrystalDetector, and finally crystal centering was completed. On average, it took 117.2 s (ten attempts) for the whole crystal centering sequence. At this stage, all calculations for the sample detection were executed on a CPU-based calculator. DeepCentering is called about ten times in the whole centering process. Also, one detection time is about 2.0 s when using CPU and less than 0.2 s when using GPU. Therefore, if a GPU machine is used for crystal centering, the centering time will be shortened by 20 s at least.

## 3. Results   

### 3.1. Cryo-loop centering using LoopDetector   

We tested 100 samples using LoopDetector to assess the cryo-loop detection accuracy. All loop detection was successful and loop centering was correctly completed as rough centering to proceed to the next step (*i.e.* crystal centering using CrystalDetector). Compared with the conventional method that utilizes background subtraction, DeepCentering does not require an update of background images or maintenance of the gain control of the digital capture device.

### 3.2. Crystal detection using CrystalDetector   

**Figure 4:**
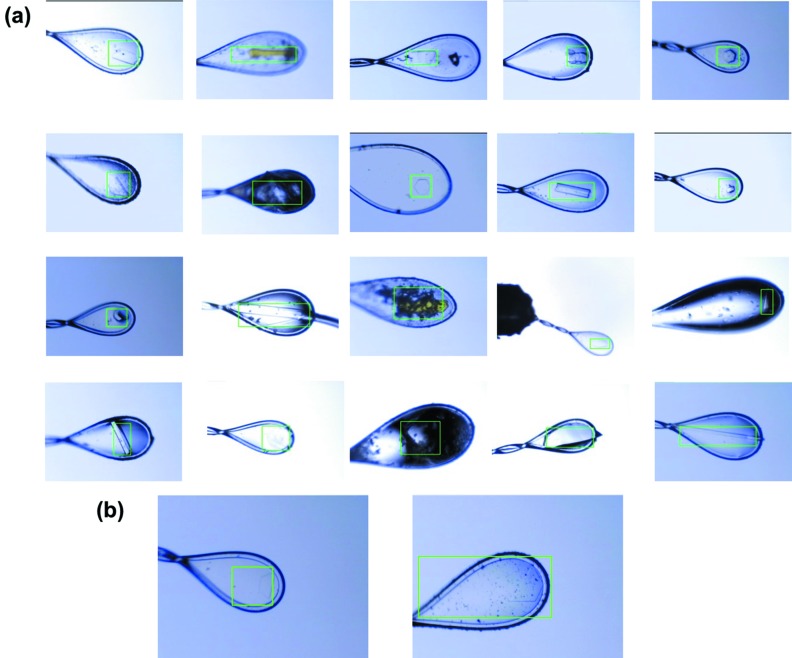
Detection results of CrystalDetector. (*a*) Successful cases, (*b*) failed cases. CrystalDetector worked correctly in almost all cases. Green bounding boxes represent detected areas of CrystalDetector and the final crystal positions are shown in the center of boundaries. The actual length of all images was 600 µm × 765 µm.

In total, 960 crystal images taken at the beamline were used for off-line evaluation of CrystalDetector. Crystal detection is considered as successful when the crystal is within 30 µm of the beam center, which corresponds to 30% of the beam size FWHM. The success rate of the test result was 90.5% (869/960 images). In many cases, the crystals were detected correctly (Fig. 4 ▸) despite the edge of the crystal being ambiguous. Typical cases in which DeepCentering failed to detect crystals included frost sticking to the cryo-loop, severely low contrast images between the crystal and the mother liquor on the cryo-loop (Fig. 4 ▸), and crystals that were invisible to the human eye.

### 3.3. Fully automated structure determination   

**Figure 5:**
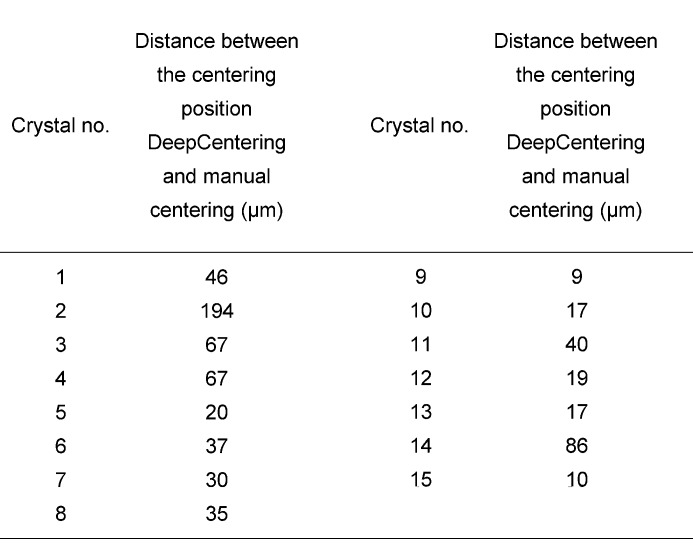
Discrepancies in the centered position with DeepCentering measured from the beam center. The distance of crystal 2 is longer than that of the others because of its large size (500 µm at the major side) and the crystal is partly shaded.

A test operation of the automated diffraction data collection and structure analysis was conducted for test samples of 15 lysozyme crystals using DeepCentering at BL26B2. Data collection and structure refinement statistics were compared with data collected from the same crystals by manual centering. The automated centering procedure for all lysozyme crystals resulted in success. All test crystals were harvested from an identical crystallization lot, and showed a similar quality to diffract up to the detector edge that corresponds to 1.58 Å. Data processing and structure refinement results also showed similar quality for all data sets on average (Fig. 2 ▸). There was no significant difference in data collection statistics between DeepCentering and manual centering experiments. Compared with the other crystals, crystals 2, 3, 4 and 6 showed poorer intensity statistics in *R*_meas_. This is probably due to the final centering position of these four crystals being slightly further from the centering position of manual centering than the other crystals (Figs. 5 ▸ and 6 ▸). As a result, the centering position of DeepCentering is in the thinner part of the crystal than that of manual centering, which makes the diffracted volume *I*/σ(*I*) in DeepCentering smaller than that of manual centering. In addition, *R*_meas_ of DeepCentering tended to show higher values compared with the manual centering values. This indicates that manual centering is more strictly centered.

However, refinement statistics for all crystals show that there was no significant difference between both centering methods. The averaged *R*_factor_/*R*_free_ values were 27.9/30.0 by DeepCentering and 28.0/29.7 by manual centering (Fig. 2 ▸). From these statistics, DeepCentering is considered to work correctly to detect the crystals, which is essential for structure determination.

## 4. Conclusions and future plans   

We have developed an automated crystal centering system using deep learning that has a high success rate of automated crystal centering. DeepCentering was applied to fully automated structure determination, including ligand screening. Since DeepCentering does not use X-rays, it might be suitable for room-temperature crystallography.

A hypothesis of promising future application of DeepCentering is *in situ* data collection using a crystallization plate scanner, which is available at one of the SPring-8 MX beamlines. Automation of series data collection for a large number of crystals in multi-well crystallization devices such as the SBS crystallization plate and micro-fluid devices, followed by the structure analysis pipeline, might be expected to accelerate the cycle of structure determination in ligand screening experiments.

DeepCentering is expected to continuously improve the accuracy of cryo-loop and crystal detection by increasing the amount of training data. There is also scope to improve object detection accuracy. Specifically, current DeepCentering outputs detection results as a boundary box (rectangle); however, an improved method, such as Mask R-CNN (He *et al.*, 2017 ▸), which outputs the object as pixels, could be used to improve the detection accuracy.

## 5. Program availability   

The object detection aspect of DeepCentering utilizes TensorFlow Object Detection API, and information and documentation can be found at https://github.com/tensorflow/models/tree/master/research/object_detection. The programming language used is Python 2.7. Other centering aspects including instrument control of the beamline utilizes BSS, the standard data collection program at SPring-8 (Ueno *et al.*, 2005 ▸). DeepCentering works using Linux CentOS 6.5 with kernel 2.6.32431. Currently, DeepCentering is available at BL26B2, SPring-8; however, it is straightforward to implement at other SPring-8 MX beamlines that apply common control systems and experimental devices. The possibility of adapting this method to other facilities should be discussed with the authors.

## Figures and Tables

**Figure 6:**
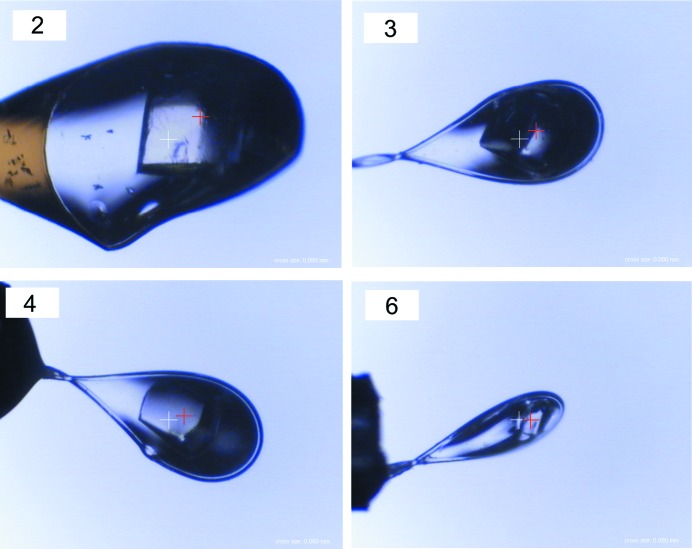
Crystals for which the centering positions from DeepCentering were slightly different from manual centering. Red and white crosses show the position of manual centering and DeepCentering, respectively. The numbers on the top left correspond to the crystal numbers in Fig. 5 ▸.
